# The Cause of Cholera

**Published:** 1887-01

**Authors:** 


### THE CAUSE OF CHOLERA.

Dr. E. O. Shakespeare, of Philadelphia, was sent by the United States authorities to investigate the recent epidemic of cholera in Europe and India. Some time ago he gave a lecture upon his experience, which is briefly this: That cholera was caused by dirt and wretched hygienic surroundings, and spread by infection being carried in clothing, etc. Of the comma-bacillus as a *cause* of cholera Dr. Shakespeare does not seem to have found any proof.

In his report to the Government, a full account will be given of his investigations and the conclusions drawn from them. In the meanwhile a few extracts will be of interest.

The German and the English Commissions do not appear to have agreed upon the cause of cholera nor the necessity for quarantine. The English Commission sent out to India submitted a report in which they related observations and conclusions directly in opposition to the German Commission's deductions. After his own study of the conditions and operations of the disease, Dr. Shakespeare is forced to the conclusion that it is doubtful whether the disease is contagious in the general acceptance of the term. The question of the efficiency of quarantine was at once raised by the conflicting conclusions of these Commissions.

Dr. Shakespeare said he was struck with the cleanly appearance of Palermo. The streets were well paved and clean, and on first impression it was one of the cleanest cities in Europe. But in going into the alleys, courts, and by-ways, just off the principal streets, he was shocked by the reeking filth and abominations of every conceivable sort. The same conditions he found in all Italy, Spain, and Southern France. The houses were constructed with extremely bad sanitary arrangements. One general water well served for many families who occupied the many-storied tenement-houses. The sinks and water-closets were so close to the well as to permit of dangerous contamination, the alluvial soil allowing of great permeation. The inmates of the houses persisted that none of the water from the wells was used for drinking or cooking purposes, but for these water was obtained from the public fountains. He learned, however, that such was not true. Cholera was introduced into Palermo from Marseilles by a man who purchased some clothing from an infected person. He was employed on a vessel, and took the clothing ashore in the night. He lived in a court. His wife washed the clothing at one of the public tubs in the centre of the court, which, through faulty drainage, leaked into the well. In a few days the man's wife was seized with cholera, his child died of it, and in a very short time nearly the whole court was infected.

When he reached Spain, the Doctor said he found a system of irrigation left by the Moors that must certainly be the source of the spread of the cholera. He had seen in the numerous canals women washing clothes, and from the same stream, very near them, others carrying water for drinking and household purposes.

The Doctor exhibited by a large map a peculiar condition of affairs in India, where cholera is epidemic. The map showed a suburb of Calcutta, which lies on low ground at the delta of the Ganges River. In building and throwing up earth for their buildings citizens constructed what are called "tanks" all over the city. These are filled by the rains of the monsoon season, the water reeking with filth, except in the public "tanks," from one monsoon season to another. He had seen women come down with their children and bathe in the water, while right at their elbow a neighbor brought a dish of food and washed it in the same water. Clothes are washed in the tanks, and they are used for all purposes, people coming from all parts of the city to use them.

The Doctor gave an interesting account of his investigations of Ferran's inoculations at Valencia. He said that in one village, out of eight thousand and forty-one not inoculated, five hundred and sixty-one were attacked and thirty-four died, while out of one thousand four hundred and nineteen inoculated forty were attacked and seven died. "My investigations of comma-bacillus," he said, in concluding, "leave me in doubt as to its being the cause of the disease. It is not a matter of the greatest importance whether it is the cause. I think Koch has conferred upon humanity a benefit of incalculable value—the placing in the hands of the physician of even ordinary intelligence the means of knowing whether, in the first suspicious case in a village, he has to deal with Asiatic cholera or ordinary cholera morbus."

